# Taking Lessons from CAR-T Cells and Going Beyond: Tailoring Design and Signaling for CAR-NK Cells in Cancer Therapy

**DOI:** 10.3389/fimmu.2022.822298

**Published:** 2022-03-18

**Authors:** Katharina Eva Ruppel, Stephan Fricke, Ulrike Köhl, Dominik Schmiedel

**Affiliations:** ^1^ Fraunhofer Institute for Cell Therapy and Immunology (IZI), Department for GMP Process Development & ATMP Design, Leipzig, Germany; ^2^ Fraunhofer Institute for Cell Therapy and Immunology (IZI), Leipzig, Germany; ^3^ Institute for Clinical Immunology, University of Leipzig, Leipzig, Germany; ^4^ Institute of Cellular Therapeutics, Hannover Medical School, Hannover, Germany

**Keywords:** chimeric antigen receptor (CAR), CAR-NK cell, cell signaling, gene therapy, CAR-T cells, allogenic cell therapy, NK cell receptors and ligands, immunotherapy

## Abstract

Cancer immunotherapies utilize the capabilities of the immune system to efficiently target malignant cells. In recent years, chimeric antigen receptor (CAR) equipped T cells showed promising results against B cell lymphomas. Autologous CAR-T cells require patient-specific manufacturing and thus extensive production facilities, resulting in high priced therapies. Along with potentially severe side effects, these are the major drawbacks of CAR-T cells therapies. Natural Killer (NK) cells pose an alternative for CAR equipped immune cells. Since NK cells can be safely transferred from healthy donors to cancer patients, they present a suitable platform for an allogeneic “off-the-shelf” immunotherapy. However, administration of activated NK cells in cancer therapy has until now shown poor anti-cancer responses, especially in solid tumors. Genetic modifications such as CARs promise to enhance recognition of tumor cells, thereby increasing anti-tumor effects and improving clinical efficacy. Although the cell biology of T and NK cells deviates in many aspects, the development of CAR-NK cells frequently follows within the footsteps of CAR-T cells, meaning that T cell technologies are simply adopted to NK cells. In this review, we underline the unique properties of NK cells and their potential in CAR therapies. First, we summarize the characteristics of NK cell biology with a focus on signaling, a fine-tuned interaction of activating and inhibitory receptors. We then discuss why tailored NK cell-specific CAR designs promise superior efficacy compared to designs developed for T cells. We summarize current findings and developments in the CAR-NK landscape: different CAR formats and modifications to optimize signaling, to target a broader pool of antigens or to increase *in vivo* persistence. Finally, we address challenges beyond NK cell engineering, including expansion and manufacturing, that need to be addressed to pave the way for CAR-NK therapies from the bench to the clinics.

## CAR-Based Immunotherapies

Cancer immunotherapies utilize the capabilities of the immune system to efficiently target tumor cells. In recent years, T cells equipped with a chimeric antigen receptor (CAR) showed promising results against B cell malignancies, proving to be a possibly curative treatment option (1, 2). CARs are artificial proteins that are composed of an antibody-derived extracellular target-binding domain, predominantly of a single chain variable fragment (scFv), that is connected to an intracellular signaling domain by a hinge region and a transmembrane domain. Upon binding, e.g., to tumor-specific or -associated antigens, intracellular CAR signaling is activated, resulting in cytotoxicity of the CAR-modified immune cell towards the target cell as well as cytokine release (3).

As of 2021, five CAR-T cell products have been approved by FDA and EMA. With BCMA (B cell maturation antigen), a second target antigen in addition to CD19 has been granted market access (1). Therefore, CAR immunotherapies are now available for the treatment of multiple myeloma and specific B cell malignancies (2). The steadily growing number of clinical CAR-T cell trials enables the treatment of additional tumor indications. All currently approved CAR-T cell products are autologous medications, which require patient-individual manufacturing. This process is complex and requires suitable production facilities, leading to high prices. Additionally, the quality of autologous CAR-T cell products is often affected by the heavy pre-treatment of patients, leading to production failure. Especially in solid tumors, CAR-T cells frequently lack clinical efficacy, caused by cellular exhaustion that can result in therapy failure (4). These factors pose major hurdles for the implementation of future widely applicable CAR therapies. Other drawbacks of CAR-T cell therapies are possibly severe side effects, e.g. cytokine release syndrome (CRS) or neurotoxicities (1). An additional risk when modifying cancer patient’s immune cells is described in a case report: During the manufacturing process of autologous, CD19-specific CAR-T cells, the CAR transgene was mistakenly introduced into a single leukemic B cell resulting in relapse with a B cell lymphoma clone resistant to CAR-T cell therapy (5).

To overcome the above stated limitations and to generate more standardized, cost-effective immunotherapies against cancer, strategies to generate CAR-T cells that are allogeneically applicable are currently developed (6). In parallel, several approaches are explored to use immune cells of healthy, allogeneic donors as a source for immunotherapies. To this end, different immune cell types are tested, especially focusing on Natural Killer (NK) cells, as these immune cells, unlike T cells, do not cause graft versus host disease (GvHD), even if donor and recipient have an HLA mismatch (7).

## NK Cells in Immunotherapy – a Revisited Cell Therapy

NK cells pose an interesting alternative to T cells for the generation of CAR equipped cells as an “off the shelf” cell therapy due to their potential to be safe and adoptively transferred from healthy donors to cancer patients. For over twenty years, NK cells have been used as adoptive therapy both in tumors of hematological and solid origin (8, 9). NK cells, collected either from patients or healthy donors, are predominantly activated and expanded *ex vivo* using cytokines like IL-2, IL-15, IL-12, IL-18, IL-21 or combinations thereof, and then infused to the patient (10–12). Additionally, in some clinical settings, patients were subjected to cytokines like IL-2 after DLI (donor lymphocyte infusion) in order to extend NK cell life-span and cellular activity *in vivo*. Several studies showed that NK cells can be safely applied in virtually unlimited numbers without the appearance of major side effects (8, 9). In some studies, NK cell infusions to patients recovering after hematopoietic stem cell transplantation showed beneficial effects (13). The overall clinical benefit for patients suffering from tumors of detectable burden, however, is fairly limited in most studies, likely due to poor recognition of tumor cells and quick exhaustion of NK cells in the immuno-suppressive tumor microenvironment (TME) (9, 14).

The great clinical success of CAR-T cells is a stepping stone for the development of next generation NK cell therapies. Using artificial receptors to recognize tumor cells, the limited efficacy of NK cells can be overcome, while retaining the favorable safety properties of NK cells when used as an allogeneic “off-the-shelf” therapy. Therefore, CAR-NK cells have been generated and assessed for efficacy in many pre-clinical and clinical studies, suggesting similar clinical benefits using CAR-NK cells as compared to CAR-T cells (8).

## T and NK cells - Two Cytotoxic Lymphocytes Differently Triggered

NK and T cells are both lymphocytes with cytotoxic or regulatory functions. When encountering and killing target cells, both exhibit similar mechanisms of action (15). NK cells can induce cell death by activating death receptors like FAS or TRAIL, but the predominant mode of action is the release of perforin and granzyme B to induce cellular apoptosis (16, 17), also utilized in CAR-mediated cytotoxicity.

In T cells, TCR signaling is the only genuine activating signal and an ultimate requirement to trigger cytotoxicity. Additionally, co-stimulatory signals exist that significantly modify the T cell response when triggered simultaneously with the TCR, such as CD28, 4-1BB, OX-40 or NKG2D (18, 19). These costimulatory signals prevent cellular exhaustion, or stimulate persistence, proliferation, differentiation or cytokine secretion, respectively. In consequence, clinically used CAR-T designs consist of a signaling domain assembled from CD3ζ signaling motifs (that mimic TCR signaling) and a single co-stimulatory domain (CD28 in Yescarta^®^ and Tecartus™; 4-1BB in Kymriah^®^, Breyanzi^®^ and Abecma^®^) (2). In preclinical studies, additional formats that enhance functionality or safety are being tested, e.g. the combination of several co-stimulatory domains, the effects of artificial, CAR signaling-dependent promoter elements or logic gated CAR expression (20–24).

**Figure 1:**
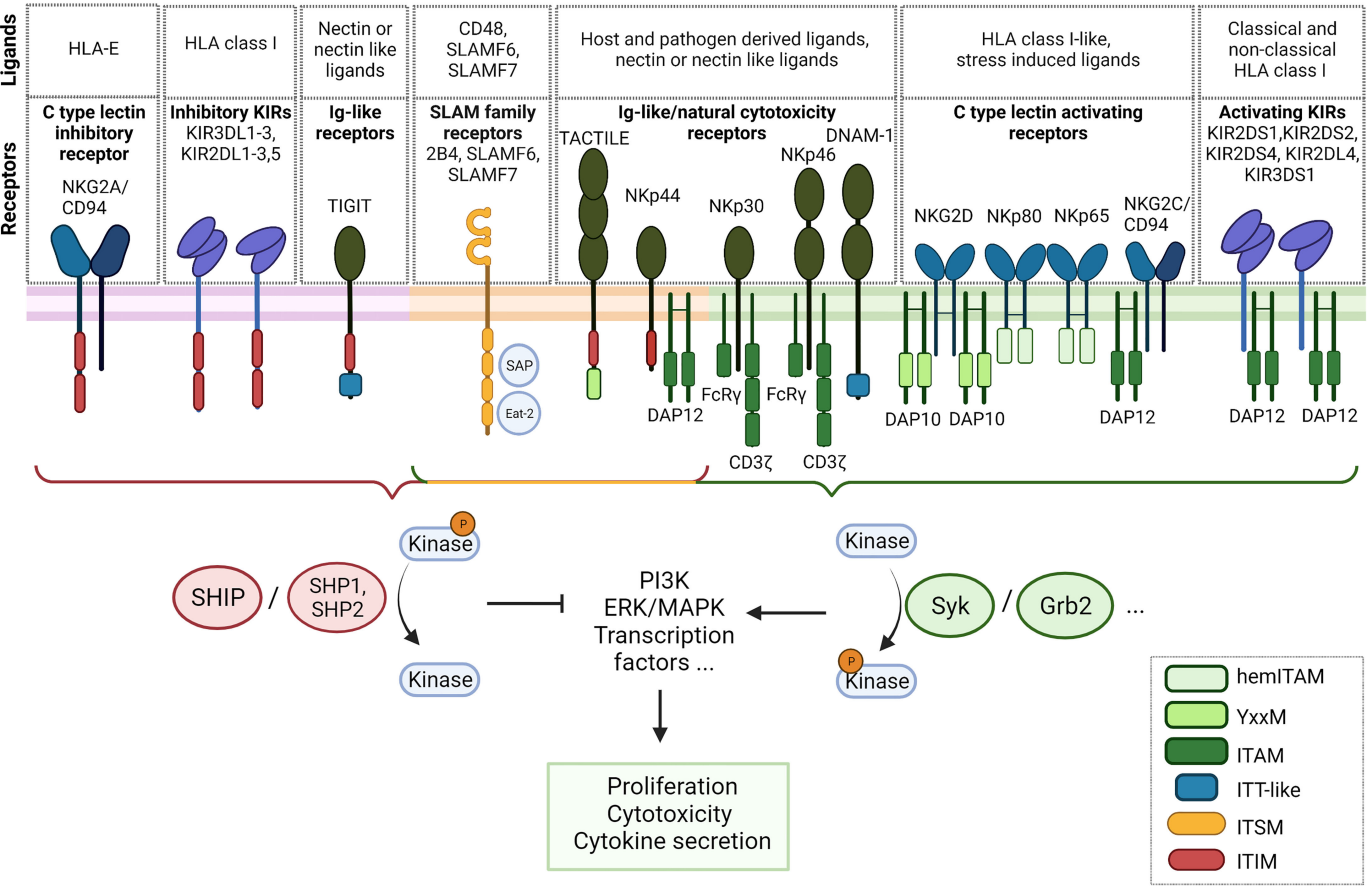
Overview of NK cell receptors and their signaling adapters. Inhibitory receptors are situated on the left (violet membrane), receptors with activating and inhibitory signaling motifs central (orange membrane) and activating receptors on the right (green membrane). ITAM, immunoreceptor tyrosine-based activation motif; hemITAM, hemi-immunoreceptor tyrosine-based activation motif; ITSM, immunoreceptor tyrosine based switch motifs; ITIM, immunoreceptor tyrosine-based inhibitory motif; ITT, immunoglobulin tail tyrosine.

NK cells encode a vast array of both activating and inhibitory receptors which are, compared to the clonotypic TCR, germline encoded (**Figure 1**). There are several activating receptors that activate NK cells upon recognition of ligands on target cells, e.g. virus infected cells or tumor cells. Diverse co-stimulatory receptors distinctively guide the NK cell gene expression profile and fine-tune their activation (25). Thereby, the extent of signaling of each individual receptor is taken into account and the multitude of integrated signals determines NK cell behavior (25, 26). On top, the receptor expression of both activating and inhibitory depends on NK cell residency in tissues, is dynamic and surprisingly diverse on a single cell level (27–29). Depending on cellular activation status, exposure to ligands or cytokines, different receptors are being expressed. The fine-tuned interaction of the receptors with inhibitory and activating signals determines whether NK cells initiate cytotoxicity, cytokine secretion or proliferation, or remain tolerant. Molecularly, upon ligand binding, both activating and inhibitory signaling motifs are phosphorylated by Src family kinases (30). Subsequently, activating receptors recruit kinases which trigger phosphorylation cascades when activated, whereas inhibitory receptors recruit and activate phosphatases which counteract activating signaling by removing phosphorylations, thereby in turn interrupting these cascades. Therefore, the balance of kinase and phosphatase activities determines NK cell tolerance and activation.

## NK Cell Signaling - a Fine Tuned Interaction of Activating and Inhibitory Receptors

The repertoire of NK cell receptors can be clustered into different families. The killer cell immunoglobulin-like receptors (KIRs) comprise both activating and inhibitory receptors which predominantly bind to HLA-A, -B and -C (26, 31). The nomenclature of KIRs is derived from the length of the cytoplasmic tail. Inhibitory KIRs have a long cytoplasmic tail with an immunoreceptor tyrosine-based inhibitory motif (ITIM) (31). Upon binding to their respective ligands, phosphorylation of the ITIM motif leads to the recruitment of the Src homology (SH) 2 domain-containing protein tyrosine phosphatases SHP-1 and SHP-2 and the inositol phosphatase SHIP. These downstream phosphatases dephosphorylate phosphorylated members of activating signaling cascades. SHIP, e.g., dephosphorylates the second messenger PIP3 within the PI3K pathway, a central signaling pathway that is crucial for NK cell cytotoxicity (31–33). In contrast, activating KIRs generally have a short cytoplasmic domain and lack a signaling domain. However, by means of a positively charged amino acid residue in their transmembrane domain, they recruit the activating signaling adapter DAP12 containing immunoreceptor tyrosine-based activation motifs (ITAM) that leads to the subsequent activation of Syk and PI3K (31, 34). Inhibitory and activating KIRs can share the same ligand, e.g. activating KIR2DS1 and inhibitory KIR2DL1 both bind to HLA-C2 (31). Additionally, some KIRs recognize non-classical HLA class I molecules, such as HLA-G, recognized by KIR2DL4 (35), or HLA-F, recognized by KIR3DS1 (36). Recently, HHLA2, a non-HLA ligand, has been described to interact with the inhibitory receptor KIR3DL3, an interaction suggested as a potential target for immune checkpoint inhibition (37, 38).

Inhibitory KIRs are considered the major mediator to maintain tolerance towards healthy tissues by sensing HLA class I, along with the inhibitory receptor NKG2A (39). NKG2A belongs to the C-type lectin superfamily that also comprises inhibitory and activating receptors (40). NKG2A, as well as its activating counterpart NKG2C, forms heterodimers with CD94 and recognizes the non-classical HLA class I molecule HLA-E. The intracellular tail of NKG2A contains two ITIM motifs to recruit phosphatases that inhibit NK cell signaling. Interestingly, in the sequence of NKG2C, both relevant tyrosine residues are mutated, therefore the ITIM motifs are dysfunctional. Instead, the NKG2C receptor recruits DAP12 to induce an activating signal *via* Syk (41). The homodimeric activating receptor NKG2D can recruit the DAP10 signaling adapter that contains a YxxM motif which signals *via* PI3K (42). This receptor recognizes a family of HLA class I-like, stress-induced ligands, named MICA, MICB and ULBP1-6, that frequently appear on the surface of tumor cells, and is critically involved in immune-surveillance (43). Two additional lectin-like NK cell receptors are NKp80, which recognizes AICL, and NKp65 which binds KACL. Both receptors encode a modified ITAM motif with reduced signaling capacity termed hemi-immunoreceptor tyrosine-based activation motif (hemITAM) (44, 45).

A group of activating Ig-like receptors termed natural cytotoxicity receptors (NCR) plays an important role in the recognition of both tumor cells and virus infected cells. The family comprises the three type I transmembrane receptors NKp30, NKp44 and NKp46. NCRs recognize heterogenous host- and pathogen-derived ligands. NKp46 and NKp30 recruit the activating downstream adaptors CD3ζ or FcRγ. Interestingly, different isoforms of the NKp44 have shown to transduce either inhibitory signals *via* a cytoplasmic ITIM motiv, or activating signals by associating with the downstream adaptor protein DAP12 (46, 47). Of note, NK cells encode multiple so-called paired receptors (48). These receptors are either activating or inhibitory but share the same ligands, usually with the inhibitory receptor possessing higher affinity and therefore being dominant when expressed at the same time. An example for these paired receptors pose the Ig superfamily receptors DNAM-1, TIGIT, CD112R/PVRIG and CD96/TACTILE which bind to tumor associated ligands of the Nectin or Nectin-like (Necl) family. Binding of DNAM-1 to its ligands such as CD155 promotes the cytotoxicity of NK cells against a range of tumor cells, mediated by an immunoglobulin tail tyrosine (ITT)-like motif with downstream Grb2 signaling (49). The inhibitory receptors TIGIT, CD112R and TACTILE act suppressive when binding to the same family of ligands (50). CD112R contains an inhibitory ITIM consensus sequence. TIGIT possesses both an inhibitory ITIM and an ITT-like motif that directly interacts with downstream regulators, e.g. SHIP1 and Grb2 (51, 52). The role of TACTILE remains unclear as it contains both an inhibitory ITIM motif and an YxxM motif that is found on various activating receptors (53, 54).

NK cells have three signaling lymphocytic activation molecule (SLAM) receptors, namely 2B4/SLAMF4, NTB-A/SLAMF6 and CRACC/SLAMF7 (55, 56). These Ig-like receptors play an important role in fine tuning cytotoxic responses of NK cells. Whereas SLAMF4 binds to CD48, SLAMF6 and SLAMF7 bind themselves in a homophilic manner in cis and trans (55, 56). They act activating, upon phosphorylation of the cytoplasmic immunoreceptor tyrosine based switch motifs (ITSMs) and the downstream adaptors SLAM-associated protein (SAP) and Ewing sarcoma-associated transcript (EAT-2). SLAMF6 can additionally act inhibitory upon downstream recruitment of SHP-1, SHP-2 and SHIP1 phosphatases that compete with SAP for the same, phosphorylated ITSMs (55, 57–60).

There are additional NK cell specific receptors like KLRG1 (61) and Siglec family of receptors (62) which are not described here. The repertoire of NK cell receptors is very complex and few synergistic effects between receptors have been described (63–65). However, research on (novel) receptors, ligands and functional mechanisms to better understand NK biology, but also their potential for immunotherapy, is still ongoing. The balance of activation or inhibition of NK cells *via* phosphorylation by kinases and dephosphorylation by phosphatases is a fine-tuned process with some variables still under investigation (26, 66–68). Therefore, the exact transcriptional changes induced in NK cells when encountering a target cell are barely predictable using our current knowledge about potential additive, synergistic or counteracting signaling pathways. A systematic assessment of the signaling pathways with a particular focus on the interplay of activating signals is still lacking, and CAR technology may provide a suitable means of addressing such open questions in NK cell biology.

## NK Cell Tailored CAR Designs Promise Great Potential to Enhance Immunotherapies

**Figure 2:**
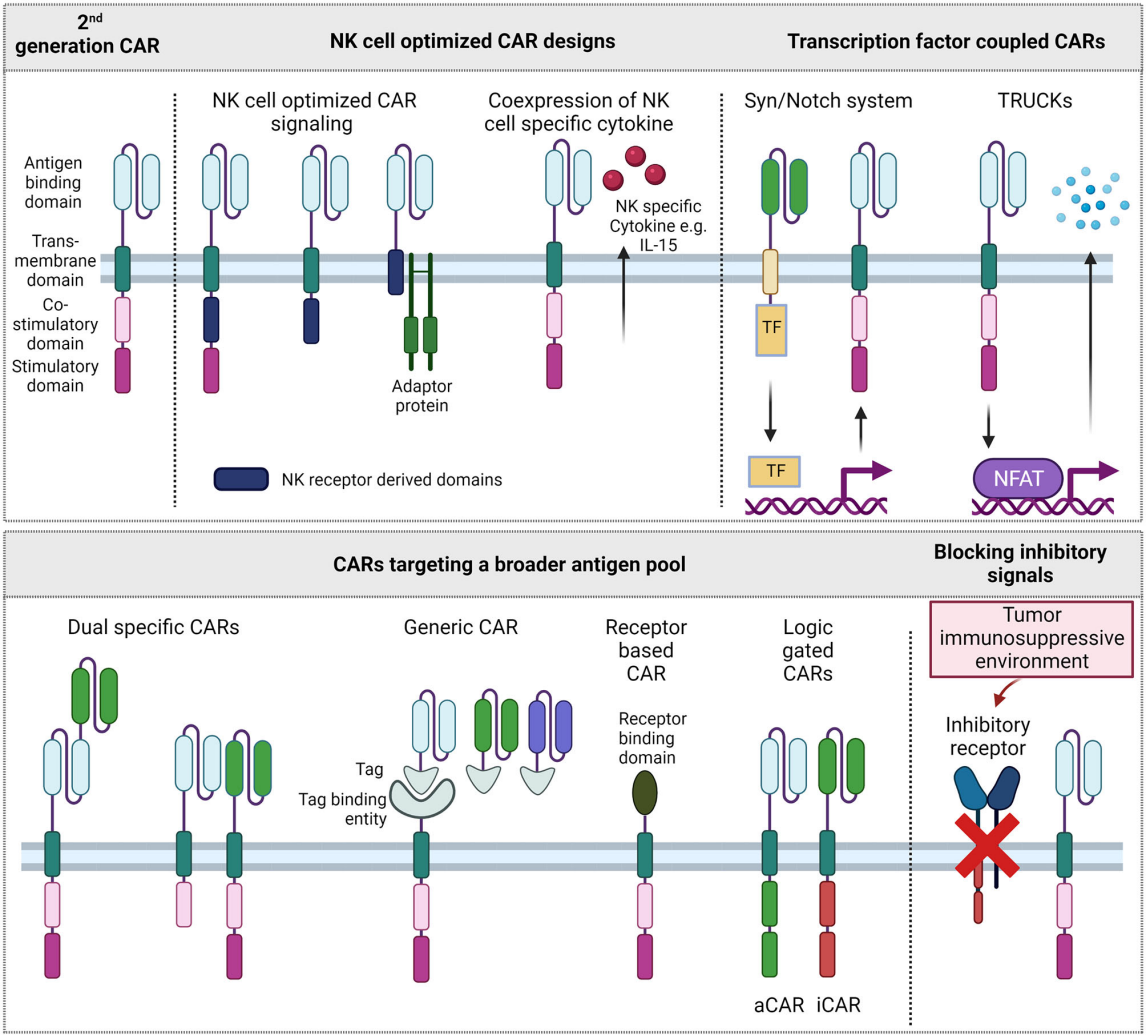
Different CAR formats. Upper left: 2nd generation CAR format that is approved on the market for T cells. Other CAR designs improve signaling for NK cells, couple CAR expression and activation to transcription factor systems, target a broader pool of antigens or aim to overcome inhibitory signals of the tumor immunosuppressive environment. TF, Transcription factor; aCAR, activating CAR; iCAR, inhibitory CAR.

Although the cell biology of T and NK cells deviates dramatically in terms of signaling acquisition and integration, the development of CAR-NK cells follows frequently within the footsteps of CAR-T cells, and T cell technologies are simply adopted for NK cells. Accordingly, in the first published clinical trial of CAR-NK cells, the CAR signaling domains are derived from the TCR and 4-1BB like used for CAR-T cells. These T cell based CAR constructs showed promising efficiency against B cell malignancies (69). The large repertoire of NK cell activating receptors and adaptor proteins, however, provides a pool of signaling domains that might improve CAR signaling responses for NK cells (**Figure 2**).

Among the many NK cell activating receptor and adaptor protein domains that could be explored as CAR signaling domains, the signaling domains of the activating receptor 2B4, and the adaptor proteins DAP10 and DAP12 have shown to induce superior cytotoxic activity of CAR-NK cells towards their respective targets. Two early studies explored the CAR constructs with a DAP12 derived signaling domain in the NK cell line YTS and showed potent cytotoxicity towards target cells (70, 71). In a study targeting Mesothelin, combinations of costimulatory domains upstream of a CD3ζ domain were screened. Notably, in addition to intracellular signaling parts, NK cell receptor derived transmembrane domains CD16, NKp46, NKp44 and NKG2D were included in the screening. As transmembrane domains of several activating NK cell receptors, like KIR2DS1, NKp46, NKG2D, possess a charged amino acid in their transmembrane region capable of directly interacting with downstream signaling adapters (72–74), CAR constructs including an NKG2D transmembrane domain as well as DAP10 and 2B4 costimulatory domain were identified as the most promising candidate to increase CAR-NK cell mediated cytotoxicity (75). A recent study explored DNAM1- and 2B4-derived costimulatory domains in CAR constructs in a combination with CD3ζ for NK-92 cells, a NK cell line with cytotoxic abilities, showing that the NK cell-specific costimulatory signal increased the persistency, proliferation and cytotoxicity of CAR-NK92 cells compared to CD3ζ alone or combined with CD28 signaling (76).

In an alternative approach, the design of the CAR construct is solely based on the NK cell specific receptor KIR2DS4 as transmembrane and intracellular domain, without a fused signaling adapter (77). Instead, KIR2DS4 adaptor protein DAP12 is co-expressed with the CAR, separated by a P2A site. Although DAP12 in this case is expressed independent of CAR target binding, the CAR-NK cells show a specific cytotoxicity against target tumor cells. The CAR in this study is directed against HLA-G, an antigen that is expressed on numerous solid tumors but restricted on healthy tissues, mostly in immune-privileged sites (77). Generally, finding suitable antigen targets for CAR therapies is crucial as targets need to be specific for the tumor while not being expressed on healthy tissue. Multiple novel targets are tested for CAR therapies, mostly on T cells, results however might be applied to NK cells (78). As single and tumor specific targets are hard to find, binding domains of receptors targeting ligands on diseased cells can be used to target a broader spectrum of antigens. Therefore, some CAR constructs exploit and enhance the natural tumor recognition of NK cells. NKG2D can bind up to eight stress induced ligands that are frequently upregulated in tumor cells and less expressed on healthy tissues. NKG2D-based CAR-NK cells have been shown effective against multiple myeloma (MM) cells in a preclinical setting (79), in a clinical study with colorectal cancer patients (80) and in various other studies using a NKG2D construct in T cells. Similarly, multiple tumors were targeted with NK cell receptor based NKp44 or NKp46 -CAR in T cells (81, 82).

By using two or more antigen directed scFvs within CAR cells, the spectrum of targets can be broadened further. These tandem or bispecific CARs combine two scFv domains within the same CAR binding domain such as in two phase I CAR-T cell studies targeting CD19 and CD22, CD19 and CD20 or BCMA and CD38. Targeting more than one antigen might thereby reduce the risk of antigen negative relapse (83–85). Alternatively, two CARs targeting different antigens can be coexpressed in the same cell. This approach was followed in a CAR-T cell study targeting CD19 and CD123 and prevented antigen negative relapses in xenograft models (86). The intracellular costimulatory and stimulatory domains of the two individually expressed CARs can either be identical or different to each other. Given the large variety of NK cell activating receptors and adaptor proteins, only few combinations of intracellular signaling adapters or domains were tested as CAR-NK signaling domains. There might be a hidden potential in assessing synergistic but also inhibiting effects. This potential has been recently tapped in NK cells by developing a logic-gated approach of an inhibitory CAR (iCAR) which will inhibit the cytotoxic signaling of an activating CAR (aCAR) upon target binding. In this approach, healthy tissue is shielded while more antigens can be explored on tumor cells (87).

In a modular approach, CAR modified cells are directed against multiple and or varying targets. An antigen targeting element, mostly scFv derived, is fused to a tag or another CAR binding property. The CAR consists of a tag or adaptor binding region and the usual signaling module. Although mostly applied in the T cell setting (88, 89), this setting has been successfully translated to NK cells (90) and, in terms of targets, this approach is rather flexible and multiple targets can be addressed at once.

A special interest should be taken to T cell malignancies with currently poor clinical outcomes. As in B cell settings, T cells express unique targets which cannot be addressed with the common autologous CAR-T cell setting due to self-targeting leading to fratricide (91). NK cells can overcome this limitation as they are negative for the two mostly used T cell markers CD3 and CD4. In a preclinical study, anti CD3-CAR-NK-92 cells significantly prolonged survival of mice challenged with Jurkat cells line (92).

CAR therapies are restricted to surface antigens. In contrast, T cell receptor (TCR) recognizes degraded intracellular proteins presented by MHC. In TCR based cell therapies, effector cells express an artificially designed high-affinity TCR (93). Although TCR-based cell therapies are also mostly explored on the T cell platform, the feasibility of TCR NK cells in the NK-92 model was previously shown (94, 95).

Besides addressing more targets, CAR design can also focus on enhancing immune responses of NK cells. A limitation of NK cell therapies is the short lifetime and persistence *in vivo* (96). The integration of autocrine growth factor IL-15 as a downstream IL-15 cassette has shown increased life span and persistence in the first published clinical CAR-NK study (69, 97). An inducible MyD88/CD40 protein switch in CAR-NK cells, which lead to increased cytokine secretion upon activation and further synergistic effects with transgenic IL-15 was previously reported. A different approach to activate NK cells with IL-15 is taken in the clinical trial NKX101 where a membrane bound IL-15 is tested to increase persistence of NKG2D-OX40-CD3ζ CAR-NK cells (97). To safeguard from potential toxicity of engineered CAR-NK cells, an inducible caspase was included in addition to the CAR in several studies (69, 98).

Especially in solid tumors, the TME is immunosuppressive, due to limited supply of nutrients, decreased levels of oxygen and an accumulation of inhibitory molecules and cells. Overcoming but also exploiting and redirecting this suppressive environment is key to combating solid malignancies. Based on the success of checkpoint inhibitor therapies, making CAR immune cells more resistant to inhibitory signals is also strived for. A reduced surface expression of the immune checkpoint receptor NKG2A showed increased cytotoxicity against HLA-E-expressing tumor cells (99). The blockage of another immune checkpoint in NK cells, TIGIT, prevented NK cell exhaustion (99, 100). Another negative regulator of IL-15 signaling is the cytokine-inducible Src homology 2–containing (CIS) protein. Genetic knockout of its gene, CISH, *via* CRISPR-Cas9 showed enhanced fitness of NK cells that additionally expressed IL-15 and a CD19 specific CAR (101). Blocking of inhibitory cytokine sensing receptors such as the of the TGF-β receptor TGF-βR2 has increased the resistance of NK cells under TGF-β inhibitory conditions (102). In a different approach, the immune inhibition is reversed by fusing the exodomain of inhibitory cytokine sensing receptors to an activating endodomain or CAR signaling domain. These chimeric cytokine receptors rather enhance than inhibit the antitumoral response of effector cells within an inhibitory tumor microenvironment (103, 104).

Immune response modulating capacities are further exploited in T cells redirected for antigen-unrestricted cytokine-initiated killing (TRUCKs) (105). In the often so called 4th generation of CAR-T cells, CARs are modified to express transgenic cytokines upon CAR stimulation: Antigen binding and resulting downstream phosphorylation cascades *via* the intracellular CAR signaling domain also phosphorylate NFAT transcription factor which can subsequently bind to NFAT response elements. In TRUCK design, stimulatory cytokines such as IL-7, IL-12, IL-15, IL-18, IL-23 are genetically introduced after NFAT response elements and their induced expression at the tumor site promotes on site effect, e.g. activating other immune cells (105, 106). Another transcription factor driven system is the SynNotch System. In a CAR setting this system can be applied to as in TRUCKs to induce expression of cytokines like IL-12 (107). However, the expression of various other (effector) proteins is imaginable and currently explored, possibly overcoming some issues of finding tumor specific antigens. A system in which a SynNotch receptor first specifically binds to neoantigens EGFRvIII which induces the expression of tandem α-EphA2/IL13Rα2 CAR was previously described. This system shows high antitumor activity with restricted off-target effects and the opportunity to target cancer-associated but not completely tumor specific antigens (22).

In summary, there are many recent innovations in CAR design, with emphasis to broaden the pool of addressable antigens, and to enhance safety or efficiency of CAR cells. Many methods initially developed in the CAR-T cell context are now utilized in NK cells. However, to further enhance CAR-NK cell tumor cell killing and *in vivo* persistence, the latest CAR-based technologies developed in the T cell context (e.g. TRUCKS) need to be translated to CAR-NK cells as well, and by acknowledging NK cell characteristics in terms of activation, or by selecting the most suitable cytokines for overexpression, the chances are best to succeed. The application and development of CAR innovations on the NK cell platform is still a large playground with a lot of potential to overcome the limitations of the CAR-T cell market.

## Challenges in NK Cell Based Therapies

Signaling enhancement and tailored adaptation of the CAR construct for NK cells constitutes just one step to success for NK-cell based immunotherapies. Deciphering critical parameters that affect *in vivo* persistence and anti-cancer efficacy of NK cells beyond the CAR-mediated effects will be critical for a clinical “off-the-shelf” product, and additional genetic modifications or combination therapies with chemo-, radio-, or immune checkpoint inhibitor therapies may be required to yield optimal results of immunotherapeutic approaches. On top, beyond biological properties and *in vivo* behavior of engineered NK cells, there are several additional technological hurdles to overcome to get NK cell technologies market-ready.

Currently, several sources for NK cells are under investigation. NK cells collected from peripheral blood (PB-NK) are easily accessible from healthy donors, but show high donor variation in terms of expansion rate, transgene-expression and efficacy, and possess overall a heterogenous and mature phenotype following expansion. NK cells derived from cord blood (CB-NK) are less mature before and after expansion, easier to engineer and easily accessible as well (108). Additionally, NK cells can be differentiated from hematopoietic stem cells (HSCs) (109, 110), induced pluripotent stem cells (iPSCs) (75) as well as from other immune cells using direct reprogramming (drNK) within a few days (111). These cells are more difficult to manufacture in research facilities due to the added complexity of differentiating NK cells in a first step, however, stem cells pose a virtually unlimited source of homogenous, highly active NK cell populations. However, if signaling and immune-suppressive factors of the TME affect NK cells of all sources in the same manner is still an open question, as most studies utilize just a single source of cells, with few exceptions (111), and side-by-side comparisons, especially under challenging conditions, are scarce. Possibly, genetic engineering has to be adjusted both to source and the anticipated tumor environment that needs to be overcome, therefore, differential CAR signaling, metabolic reprogramming or deletion of different inhibitory receptors need to be functionally assessed and predictive markers for NK cell *in vivo* efficacy established. On top, it is yet unclear if the heterogeneous, blood-derived NK cell products may perform better in patients compared to uniform, homogeneous stem cell-derived NK cells as they may react more plastic to immune suppression and possibly elicit a more varied anti-tumor response (112). Next to primary NK cells, also the cell line NK-92 is under clinical investigation (113).

Next to biological obstacles, technical hurdles still exist. NK cells are considered hard-to-engineer and hard-to-expand compared to T cells. Recently, lentiviral transductions were significantly enhanced by introducing new transduction enhancers that facilitate viral entry (114) or suppress anti-viral cellular signaling (115). On top, a novel viral envelope derived from the baboon endogenous virus (BaEV) showed superior efficacy as compared to other lentiviral envelope proteins to successfully gene-edit NK cells (116, 117) that outperforms also alpha-retroviruses previously tested (118). There is a high donor-to-donor-variation in terms of expansion rates, transduction efficiency and *in vitro* anti-tumor efficacy, however, thinking of an “off-the-shelf” CAR-NK cell therapy, a thorough donor-selection for the most suitable NK cell starting material is likely to be performed. Transposon-based DNA delivery systems are currently assessed in clinical trials for the generation of CAR-T cells as an alternative approach for long-term transgene expression (119–122). Although the occurrence of T cell lymphomas in few patients using PiggyBac transposon system mediated gene transfer poses a setback for this technology (123, 124), the transposon technology will likely be further developed and be assessed for the generation of CAR-NK cells.

To expand NK cells efficiently, feeder cell-free and feeder cell-based protocols were established. On the one hand, feeder cell-based approaches, mostly performed with genetically optimized K562 feeder cells, show considerably superior expansion rates and were successfully used for the generation of CAR-NK cells used in the only clinical phase I trial published (69, 125). On the other hand, feeder cell-free systems are still considered and widely favored for their anticipated ease in the regulatory approval processes. Additionally, feeder free processes can be performed in existing, closed (semi-)automated production lines without major adaptations required for feeder cell line cultivation and irradiation (126–129).

Lastly, storage of an NK cell based cell product is essential to serve as an “off-the-shelf” therapy which is produced in large batches for multiple patients. However, NK cells are very sensitive to cryo-induced damages. Although cytokine activated cells exhibit higher survival rates and cytotoxic capabilities than unactivated NK cells, still, important parameters that determine efficacy *in vivo*, like proliferation or migration (130), are majorly impaired following a freeze-thaw cycle. Importantly, such parameters are frequently not addressed in quality control protocols to assess quality of NK cell products (131), therefore, reasons for potential treatment failures may also be due to undetected, limited NK cell fitness. Efficient cryopreservation protocols, preferentially without the use of toxic cryoprotectant DMSO, are still under investigation (132), and need to ensure long-term product stability, safe means of transport, and enable direct infusion of the cell product into patients from cryobags.

## Future of NK Cell Based Therapies

It is widely accepted that allogeneic products will be the next step in the development of anti-cancer cell therapies, as they promise high quality standards, immediate availability, significant cost reductions and a reduced utilization of manufacturing capacities.

However, CAR-NK cell based therapies need to prove their abilities in additional clinical trials so as to show that they are at least as effective as CAR-T cell therapies which currently monopolize the market of CAR therapy. Postulated advantages of NK cells, like innate anti-cancer immunity to possibly overcome antigen escape and cancer heterogeneity (133) or a different cytokine profile that may direct other immune cells like dendritic cells (134) into the tumor site, need to prove clinical relevance. The limited efficacy in the treatment of solid tumors observed in CAR-T cells *in vivo* may pose a similar challenge for CAR-NK cells as studies on allogeneic, activated NK cells imply that they face cellular exhaustion and poor infiltration into tumor sites (14, 135–137). To overcome immune suppression in the TME, additional arming of immune cells through genetic engineering is pushed for, e.g., by genetic ablations (138, 139) or metabolic reprogramming of NK cells (140, 141).

The generation and clinical administration of CAR-T cells is widely performed, and clinical manufacturing pipelines are established and approved. In contrast, technologies required for the optimization of cellular behavior and manufacturing processes of engineered NK cells still need refinement.

Both NK cell and T cell therapies require further research to overcome their respective limitations and to turn them into efficient and affordable living anti-cancer drugs applicable beyond B cell malignancies. Therefore, the question if T cells or NK cells are to become the dominating platform for allogeneic, “off-the-shelf” CAR cell therapies remains unanswered.

## Author Contributions

KR and DS outlined, wrote, and referenced the manuscript. KR prepared the figures. SF and UK carefully edited the work. All authors contributed to the article and approved the submitted version.

## Funding

This work was supported by the Fraunhofer Internal Programs under Grant No. Attract 131-600004 (to DS).

## Conflict of Interest

The authors declare that the research was conducted in the absence of any commercial or financial relationships that could be construed as a potential conflict of interest.

## Publisher’s Note

All claims expressed in this article are solely those of the authors and do not necessarily represent those of their affiliated organizations, or those of the publisher, the editors and the reviewers. Any product that may be evaluated in this article, or claim that may be made by its manufacturer, is not guaranteed or endorsed by the publisher.
